# Enhanced Acute Muscle Activation in ALS Patients Following Liposomal Curcumin, Resveratrol, and Dutasteride Administration

**DOI:** 10.3390/ph18040497

**Published:** 2025-03-28

**Authors:** Julio Martín-Ruiz, Rosa Maset-Roig, Jordi Caplliure-Llopis, Carlos Villarón-Casales, Jorge Alarcón-Jiménez, Nieves de Bernardo, Belén Proaño, Rubén Menargues-Ramírez, Pablo Selvi-Sabater, José Enrique de la Rubia-Ortí

**Affiliations:** 1 Department of Health and Functional Evaluation, Faculty of Physical Activity and Sports Sciences, Catholic University San Vicente Mártir, 46001 Valencia, Spain; 2 Doctoral Degree School, Catholic University San Vicente Mártir, 46001 Valencia, Spain; rosa.maset@ucv.es; 3 Department of Nursing, Catholic University San Vicente Mártir, 46001 Valencia, Spain; jordi.caplliure@ucv.es; 4 Biomechanics & Physiotherapy in Sports (BIOCAPS), Faculty of Health Sciences, European University of Valencia, 46001 Valencia, Spain; carlosalberto.villaron@universidadeuropea.es; 5 Department of Physiotherapy, Catholic University San Vicente Mártir, 46001 Valencia, Spain; jorge.alarcon@ucv.es (J.A.-J.); nieves.debernardo@ucv.es (N.d.B.); 6 Department of Basic Biomedical Sciences, Catholic University of Valencia, 46001 Valencia, Spain; joseenrique.delarubi@ucv.es; 7 Nursing Department, Faculty of Health Sciences, University of Alicante, 03690 San Vicente del Raspeig, Spain; rmr23@alu.ua.es; 8 Hospital Reina Sofia, 30003 Murcia, Spain; pablo.selvi@carm.es

**Keywords:** amyotrophic lateral sclerosis, muscle activation, curcumin, liposomal resveratrol, dutasteride

## Abstract

**Introduction:** Amyotrophic lateral sclerosis (ALS) is a neurodegenerative disease characterized by loss of electrical activity and motor control at the muscular level. Therapeutic alternatives, such as the polyphenolic antioxidants curcumin and resveratrol in liposome form, or the drug dutasteride, could be effective for muscular activity. **Objective:** To measure the acute change in electrical muscle activation after administration of a combination of curcumin in liposomal form, resveratrol, and dutasteride in patients with ALS. **Materials and methods:** Patients with bulbar and spinal ALS were selected and randomly distributed into an intervention group (IG), which received an oral combination of curcumin in liposomal form/resveratrol^®^ and dutasteride for 2 months, and a control group (CG), which received a placebo. Electrical activity to determine basal muscle activation and fasciculations was measured before and after the intervention using surface electromyography of the biceps brachii (BB), triceps brachii (TB), rectus femoris (RF), and tibialis anterior (TA). Within comparisons of pre and post-muscular variations in each group were conducted. **Results:** Electrical basal activity increased only for the IG in the right (*p* = 0.05; g = −0.45) and left (*p* = 0.004; g = −0.74) hemibody muscles and also presented less variation among them after treatment in the IG. For fasciculations, there was an increase in the total activation of the upper muscles in the IG (*p* = 0.017; g = −0.86) and for the lower muscles in the CG (*p* = 0.037; g = −0.68). The pattern of muscle activation remained constant in the IG but experienced variations in the CG.

## 1. Introduction

Amyotrophic lateral sclerosis (ALS) is characterized by muscular functional deterioration linked to various musculoskeletal alterations that, among other consequences, cause falls and bone fractures [1,2]. In both the bulbar and spinal variants of the disease, there is a loss of muscle tone, exaggerated and pathological reflexes, and cramps triggered by the slightest contractions [3]. These muscle alterations can be attenuated by neuroprotective molecules that ameliorate muscle damage and atrophy, such as polyphenols including curcumin and resveratrol, or dutasteride, by increasing the levels of free testosterone (T) [4,5].

Evidence indicates that ALS patients have impaired recruitment due to the loss of motor unit potentials [6,7], a factor that accelerates the process of sarcopenia. Muscle atrophy plays an important role in disease progression and regression.

Specifically, some muscle groups are more prone to show these symptoms, as in the case of the biceps brachii, whose electrical activity increases in a representative manner and at a high frequency, predicting a faster evolution of the disease [8]. This alteration is accompanied by fasciculations, which are spontaneous and involuntary contractions relevant for diagnosing ALS in patients. These changes, which can be measured with EMG and ultrasound, are within the framework of the Golden Coast Criteria, a reference document that explains the process of ALS diagnosis, in which at least three important factors can be highlighted: (1) progressive motor impairment, (2) dysfunction in at least one body section, and (3) fasciculation potentials [9].

This characteristic was much higher (three levels more) in subjects with ALS than in unaffected individuals. The areas that usually show this feature are mainly the proximal extremities [10], which are more prevalent on the dominant side and upper limb [11] and in the form of twitching and stiffness [12]. An example is biceps musculature, which presents this characteristic 40 times more in atrophied musculature and 10 times less in healthy musculature. Others, such as the gastrocnemius, perform five times more at the beginning and gradually less over time [13].

Several techniques have been used to detect these common alterations in all subjects, although to a lesser extent in healthy subjects. Ultrasound is more effective than surface electromyography (sEMG) in the detection of fasciculations [10]. Although they do not differ significantly from each other, they are noninvasive [14,15]. However, sEMG cannot be replaced by ultrasound, since the action potentials that it is capable of detecting are lower (values of 0.39 ± 0.25 mV) and undetectable with ultrasound [16], in addition to offering a greater variety of signal analysis [17].

After diagnosis, general care of patients with ALS is essential for managing the disease process with an acceptable quality of life [18,19]. However, at the pharmacological level, it is noteworthy that a few years ago, the only one approved worldwide for ALS was riluzole, which had a very limited effect on survival [20], and other drugs such as edaravone are now available. Therefore, it is necessary to identify novel molecules that can improve disease prognosis. Some polyphenols have been shown to exhibit excellent neuroprotective properties in cell lines and animal models of ALS [20]. In terms of neuroprotective capacity, polyphenols include curcumin and resveratrol [21]. Specifically, curcumin has been shown to be a potent antioxidant and anti-inflammatory agent with the ability to modulate different signaling pathways involved in the development and severity of neurodegenerative diseases [22,23,24], as well as having a high neuroprotective capacity [25], and preventing glutamate toxicity [26].

Specifically, resveratrol in a cellular model of the disease appears to protect against mutant SOD1-mediated toxicity through the positive regulation of sirtuin 1 expression (SIRT1) [27]. Along the same lines, in an animal model of ALS, treatment with resveratrol significantly delayed the onset of the disease and preserved the function of lower and upper motor neurons, significantly increasing their survival owing to its neuroprotective activity, associated with increased expression and activation of SIRT1 and AMPK in the ventral spinal cord, which promotes the normalization of autophagic flux and increases mitochondrial biogenesis in the spinal cord SOD1 (G93A) [28].

Curcumin has also shown benefits; specifically, after administering a dose of 600 mg/day for 6 months, a deceleration in the progression of the disease is achieved [29], and in the same line, a derivative of curcumin (GT863) significantly slowed the progression of motor dysfunction in mice and substantially reduced highly aggregated SOD1 [30]. These properties explain its efficacy in mitigating skeletal muscle damage and improving function [31].

Despite these benefits, its efficacy is limited mainly because of its low availability [32]. This limitation can be overcome if it is administered in liposomal form. Resveratrol and its natural analogs have been shown to increase stability and bioavailability [33]. Similarly, the use of nanobiotechnology with curcumin improves its bioavailability [34], which may explain its efficacy as an adjunctive treatment in patients with ALS [29,35]. In short, the use of both antioxidants combined in liposome form improves bioavailability and efficacy [36,37].

In contrast, dutasteride is a drug for benign prostatic hyperplasia that reduces circulating levels of dihydrotestosterone (DHT) by inhibiting the type 1 and type 2 isoenzymes of 5 alpha-reductase, which are responsible for the conversion of testosterone to 5alpha-DHT [38]; thus, it increases serum levels of free T and maintains its plasma levels, which act on the central nervous system (CNS) [39]. Similarly, it increases free progesterone (PROG), stimulates myelin production [40,41,42], and suppresses the inflammatory response stimulated by polysaccharides in the central and peripheral nervous systems, reducing markers of inflammation and oxidation of abnormal protein aggregates [43]. In addition, dutasteride also exhibits antioxidant activity, as free T enhances the survival of human neurons and astrocytes by inhibiting the generation of oxygen free radicals (ROS) [44,45] and nitrogen free radicals (RNS), and increasing the expression of sirtuin-1 [46].

At the muscular level, an increase in serum T has a positive effect, with an increase in stair climbing power, muscle mass, and strength [47]. Additionally, in postmenopausal women, an increase in serum T has been shown to increase lean mass and muscle power [48], and in premenopausal women, it produces an increase in muscle mass and an increase in running time to exhaustion [49], which represents an improvement in physical performance [50]. As for the increase in serum PROG levels, the results are inconclusive, although it has been shown that estrogen therapy may enhance muscle mass in postmenopausal women [51,52].

Given the level of response, the spectrum of possibilities that these drugs provide, and the need for alternatives that improve the prognosis of the disease, this study aimed to assess the acute impact on electrical muscle activation of the administration of a combination of curcumin in liposomal form and resveratrol and dutasteride in patients with ALS. For this purpose, bilateral electromyographic activities of the biceps brachii (BB), triceps brachii (TB), rectus femoris (RF), and tibialis anterior (TA) were measured under basal conditions, and the resulting fasciculation level was measured at an interval of two months.

The main hypothesis is that curcumin in liposomal form, resveratrol, and dutasteride increase muscle activation and attenuate fasciculations in patients with ALS, reducing neurodegenerative symptoms and motor losses.

## 2. Results

The sample of 61 participants had a mean age of 56 years and a higher proportion of men and patients with ALS of spinal cord origin; the average value of ALSFRS-R for these patients is 29.16 ± 8.1, as shown in Table 1.

### 2.1. Baseline Records

After two months of intervention, there were different trends in basal muscular activity in each experimental group (Table 2). The total basal activity showed a slight decrease in the CG (*p* = 0.04; g = 0.01) and a significant increase, accounting for a medium-sized effect in the IG (*p* = 0.001; g = −0.65). In addition, the mean left basal hemibody increased significantly only in the IG, with almost twice the activity after the intervention, showing the largest effect size of g = −0.74. The effect size with confidence intervals for all within-comparisons (basal and fasciculations) can be found in Table A2 (Appendix A).

**Figure 1:**
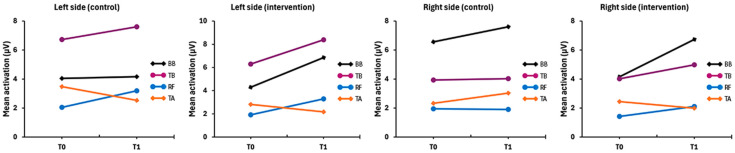
Comparison of intragroup muscle activation means between the intervention and control groups highlighted the increase in the arm muscles. Note: BB = Biceps brachii; TB = Triceps brachii; RF = Rectus femoris; TA = Tibialis anterior.

**Figure 2:**
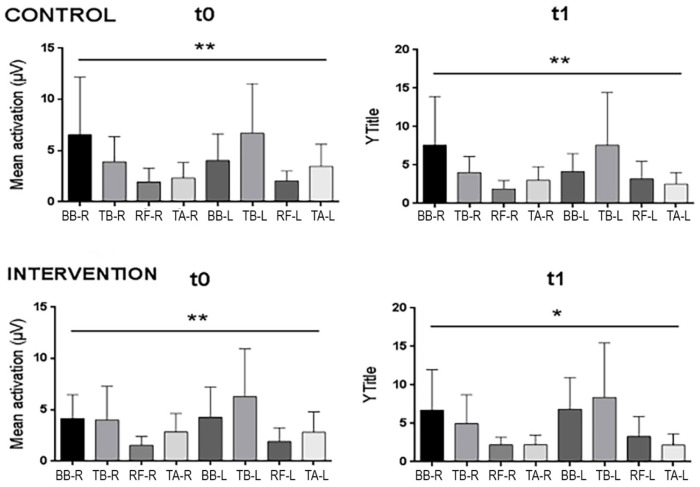
Mean basal activity of all muscle groups in the control and intervention groups at t0 and t1, with major changes in arm muscles. ** *p* < 0.01; * *p* < 0.05. Note: BB = Biceps brachii; TB = Triceps brachii; RF = Rectus femoris; TA = Tibialis anterior; L = Left; R = Right.

Electrical activity tended to increase within the IG in almost all muscle groups, which remained stable between the two measurements, with a slight increase in the BB and TB on both sides (Figure 1). These changes reflected a change in the pattern of basal activation among the muscles, as can be seen in Figure 2 (Table A1 in Appendix A), showing a more tenuous and homogeneous increase in basal activity among the muscles in the IG, with the muscles showing highly significant differences at T0 (*p* < 0.01), but then became significant only after the two-month intervention (*p* < 0.05), corroborating the previous result of higher total basal activation only in this group. In the CG, only the BB of both sides showed an increasing trend, while the other muscles remained the same or with a decreasing trend; thus, the difference among the muscles remained highly significant (*p* < 0.01) after the intervention, with the consequent slight decrease in total activity (g = 0.01) previously mentioned.

### 2.2. Fasciculations

**Figure 3:**
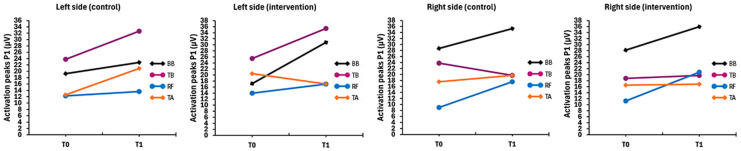
Comparison between the maximum peak muscle activation (P1) within the intervention and control groups (measurements 0 and 1) highlights the attenuation of the intervention group. Note: BB = Biceps brachii; TB = Triceps brachii; RF = Rectus femoris; TA = Tibialis anterior; P1: activation peak (maximum).

Figure 3 shows the evolution of fasciculations considering the maximum peaks (of the ten collected) of each muscle group in measurements zero and one. Slight increases were detected in the case of the left hemibody in both CG and IG, and a tendency toward moderation on the right side for both samples, with higher values in BB.

In Table 3, the activity of the fasciculations in measurements zero and one is summarized, considering the highest peak (P1), lowest peak (P10), and sum of the activation of all peaks for the upper and lower limbs. In the IG, a significant increase was observed in the lowest peak (P10) of the right BB (*p* = 0.01), highest peak (P1), and lowest peak (P10) of the left TB. Thus, the total activity of the upper limb muscles significantly increased, accounting for a large effect size (*p* = 0.007; g = −0.86). In the lower limb musculature, there was a significant increase in the highest peak of the right RA for both the CG (*p* = 0.001) and IG (*p* = 0.003), with a higher difference in the CG. In addition, the CG presented a significant increase in the lowest peak of the left RA (*p* = 0.004) and the highest peak of the left TA (*p* = 0.036), with a significant increase in total activation among all peaks of the lower limbs that accounted for a large effect size (*p* = 0.037; g = −0.68).

**Figure 4:**
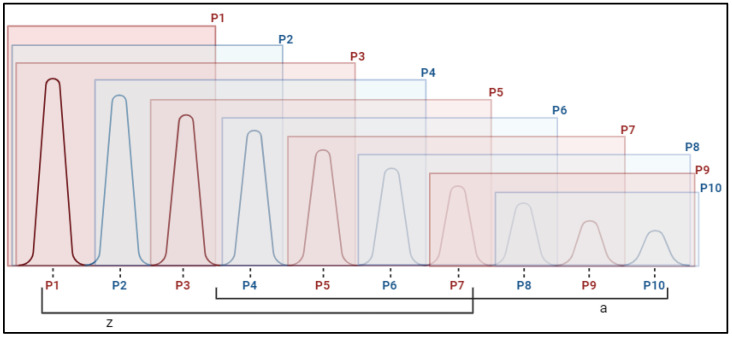
Muscle activation peaks in the muscles of ALS patients at the beginning of the study. z and a represent subgroups formed according to Dunn’s multiple comparison test. Note: P1–P10: activation peak (P1 major to P1 minor); z, a: groups of peaks of similar activation.

The activation peaks presented a symmetrical pattern in the initial measurements, in which each peak was similar (no statistically significant differences) to the next two peaks, which were larger and smaller, forming subgroups of up to five peaks. For example: P2 = P3 = P4 = P5 = P6. Consequently, peaks 1, 2, and 3 were the highest, peaks 8, 9, and 10 were the lowest, and an intermediate group of peaks 4, 5, 6, and 7 was formed, which was significantly different from the highest (P1) and lowest (P10). This pattern, depicted in Figure 4, was observed in all muscles measured in both the groups at t0.

**Figure 5:**
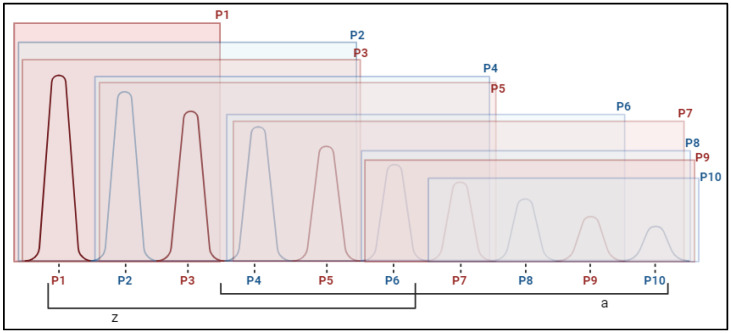
Peak muscle activation in the TA-L of ALS patients in the control group (CG) after two months of treatment. z and a represent subgroups formed according to Dunn’s multiple comparison test. Note: P1–P10: activation peak (P1 major to P1 minor); z, a: groups of peaks of similar activation.

Measurements for two months showed a similar pattern, except for the left TA of the CG, which ceased to be symmetrical, as fewer differences were found between the intermediate peaks. There are symmetrical peaks that remain similar to the two or three subsequent peaks, both higher and lower, such as P1 and P3, whereas the intermediate peaks become closer to the lower peaks, forming a group comprising the peaks from P7 to P10 (Figure 5). The most observable change is the tendency of the central peaks to become more equal to slightly lower values, attenuating spontaneous activation.

## 3. Discussion

One of the fundamental characteristics of ALS is its accompanying neurodegenerative process. In this disease, there are limited tests to diagnose and treat patients, and neurophysiological tests are fundamental [53] because they can detect downward changes in muscle tone, sarcopenia, or functional loss, objectified by a reduced contractile capacity that causes a decrease in muscle quality [54]. These changes should be reflected in muscle patterns similar to those of healthy subjects, in which there is stable activation and no spontaneous variation that could lead to loss of function.

To improve these variables, we administered a combination of curcumin and resveratrol liposomes, together with dutasteride, for 2 months. As for polyphenols, curcumin has already shown improvements in ALS patients; specifically, it has been shown to decelerate the progression of the disease, improving aerobic metabolism, and oxidative damage after administering 600 mg for 6 months [29]. Interestingly, it has already been administered in liposomal form (80 mg/day) to patients with ALS, obtaining positive results (improving the probability of survival) and demonstrating that it is safe [35]. Resveratrol has also shown promising results in animals and cellular disease [27,55]. At the cellular level, glutamate is one of the most effective molecules to counteract toxicity, which is directly related to disease pathogenesis [41]. Similarly, after the intervention with these molecules in our study, an improvement was observed in the muscle response of the IG versus the CG. The most important improvement occurred in the muscle groups of the upper limbs (BB and TB), accompanied by a slight improvement in the bilateral RF, although not as pronounced. In other words, it is more generalized and distributed among all the muscles measured, unlike the CG, where the total increase occurs because the muscles with greater baseline activity increase even more after two months. Thus, the differences between the muscle groups were less significant only in the IG group (Figure 3). These facts are in line with the studies by Neltner, Anders, Keller, Smith, Housh, Schmidt, and Johnson [56] and Alarcon-Jimenez, de la Rubia Orti, Martin Ruiz, de Bernardo, Proano and Villaron-Casales [57], in which it is evident that it is mainly in the upper muscle groups, especially in the BB, where activation changes are reflected, thus becoming the main reference for the evolution of the disease. In addition, they share the line of work of other interventions, such as therapeutic programs of moderate physical activity, specific for ALS patients with increases in muscle tone between 5 and 6% [58].

This increase in muscle activity could be explained by dutasteride, which inhibits the conversion of free (T) to dihydrotestosterone (DHT) and PROG to dihydroprogesterone (DHP), resulting in higher levels of free T and PROG. This drug has been shown to be very effective in the treatment of neurodegenerative diseases, improving muscle mass and restoring neuromuscular junction function [59,60], as evidenced by De Nicola, Meyer, Garay, Kruse, Schumacher, Guennoun and Deniselle [61]. In addition to these references, the administration of androgens facilitates an increase in muscle strength [62] and has been prescribed in anabolic therapies for the treatment of muscle wasting, and in the case of ALS patients, showing a lower level of T compared to ALS-free control groups.

On the other hand, resveratrol administration has also been linked to improvements in muscle activity and motor performance in ataxic rats [63]. In the same model, curcumin also achieved improvements of the same nature [64], suggesting that the neuroprotective effect of the two molecules may complement the action of free T and PROG hormones increased in the blood by dutasteride, thereby justifying the improvement in muscle activity observed in our study. In addition, the combination of both molecules has already produced extraordinary results in the phenotypes of fast- and slow-twitch muscles of the limbs in cachectic mice, indicating that it is a promising combination for alleviating the loss of muscle mass and dysfunction that occurs in diseases such as ALS [4].

Incidentally, the fasciculations that both groups showed at the beginning of the study underwent slight changes after two months. IG showed greater activity in two of the weakest peaks (P10) of the upper limb, right BB, and left TB, in addition to an increase in the largest peak (P1) of the left TB, with a consequent increase in the total activity of the upper limb in this group. In the CG, in contrast, there was an increase in two of the highest peaks (P1) of the lower limb, right AR and left AT, in addition to an increase in the lowest peak (P10) of the left AR and a consequent increase in the total accumulated activity of fasciculations in the lower limb. This is an interesting result that can be confirmed in future studies using ultrasound [10], although it is an advantage to use sEMG, as it is less invasive than electromyography because it is quicker and easier to perform.

These changes are associated with potential fasciculation (PFs), whose generators in ALS are active for several months, even with a significant loss of motor units, which generates unstable axonal sprouts in partial reinnervation [65]. This dynamic could explain the change in the pattern of the peaks of the left TA (Figure 5), which could be due to an increase in the lowest peak; thus, a group was formed that included greater activity for the peaks from P7 to P10 in the fasciculations experienced by the CG patients after 2 months. In IG, there was also an increase in the total accumulated activity between the peaks. In the upper limb, the pattern of peaks did not change (Figure 5) after two months, suggesting a more homogeneous increase, which is relevant because it has been demonstrated that an abrupt increase in excitability and firing frequency of the motor units is associated with disease severity [66].

Fasciculations are more common in the upper limb extremities when using ultrasound for their detection and, as has been shown in this study using sEMG, they tend to become more regular as the disease progresses. This depends on the level of neural degeneration present in each case [67], without forgetting that there are factors that can cause its alteration, such as insomnia or episodes of hyperventilation [65,68].

The time at which further neuronal degeneration and loss of activation become evident is uncertain, although the fact that there is a better basal response and activity that occurs without significant changes in the amplitude of fasciculations may give reason to believe that tasks with moderate motor control could be performed acceptably, perhaps because of better preservation of slow muscle motor neurons [69], factors that could contribute to improving the patient’s functional capacity.

The activation values recorded could be attributed to the neuroprotective effects of dutasteride [63] and the antioxidant and anti-inflammatory properties of the polyphenols, curcumin and resveratrol. In this regard, it is especially noteworthy that curcumin, in studies on animal models of spinal and bulbar muscular atrophy (a disease of the motor neurons that is often confused with ALS), produces a significant improvement in motor function in line with the results obtained in this study and increases survival time in mouse models of the disease [70].

The results of the present study are positive. However, our study has limitations that are in part related to the complexity of sample recruitment and short follow-up time. Therefore, the pattern in which these changes occur should continue to be studied to assess whether the compensatory effects that reflect greater activity in some specific muscle groups are positive. In future work, we plan to investigate whether a greater irregularity in these changes is postulated as a pathological marker in the progression of the disease with a more marked degenerative process, as observed in CG, and to monitor the stability of the changes over periods of at least six months.

However, it would be advisable to include a control group of healthy people to calibrate and compare the levels of change that occur or to include other variables that may lead to more holistic interpretations, such as bone density or heart rate variability [71]. In addition, it would be interesting to evaluate, after the intervention, the possible differences between sexes, type of ALS, and time of diagnosis on muscle activity in the medium and long term. In this regard, it would be advisable to use tools other than sEMG to perform contrast measurements, such as ultrasound, a common practice in this type of study. In addition, it is important to note that the possible influence of diet followed by the study participants on the observed changes was not assessed. This factor, and the different functional capacities of the patients, are elements to be considered in the future as they are variables susceptible to altering some of the results.

## 4. Materials and Methods

### 4.1. Study Design

A randomized, double-blind, prospective, experimental, analytical, quantitative clinical study was conducted on a sample of patients with bulbar and spinal cord ALS.

### 4.2. Participants

The sample consisted of 61 participants (men and women) diagnosed with medullary and bulbar ALS, 27 in the control group (CG), and 34 in the intervention group (IG). For patient recruitment, the main ALS centers at the state level were contacted, and after being informed of the nature of the project by means of a patient information sheet, they signed an informed consent form if they agreed to participate. The selection criteria were then applied. The inclusion criteria were as follows: men older than 18 years, women older than 18 years or between 18 and 50 years of age, patients diagnosed with ALS at least six months before the start of the study, and patients treated with riluzole. The exclusion criteria were as follows: tracheotomy, invasive or noninvasive ventilation with positive ventilatory pressure, participation in another trial or in the 4 weeks prior to inclusion, patients with evidence of dementia, alcohol or drug abuse dependence, patients infected with hepatitis B or C, human immunodeficiency virus-positive, renal patients with creatinine 2 times above normal markers 30 days prior to inclusion, and liver patients with liver markers (ALT and AST) elevated 3 times above normal levels. All the participants signed an informed consent form before the start of the study. Patients were recruited by random sampling through the ALS national associations as part of the clinical trial NCT04654689.

### 4.3. Procedure

**Figure 6:**
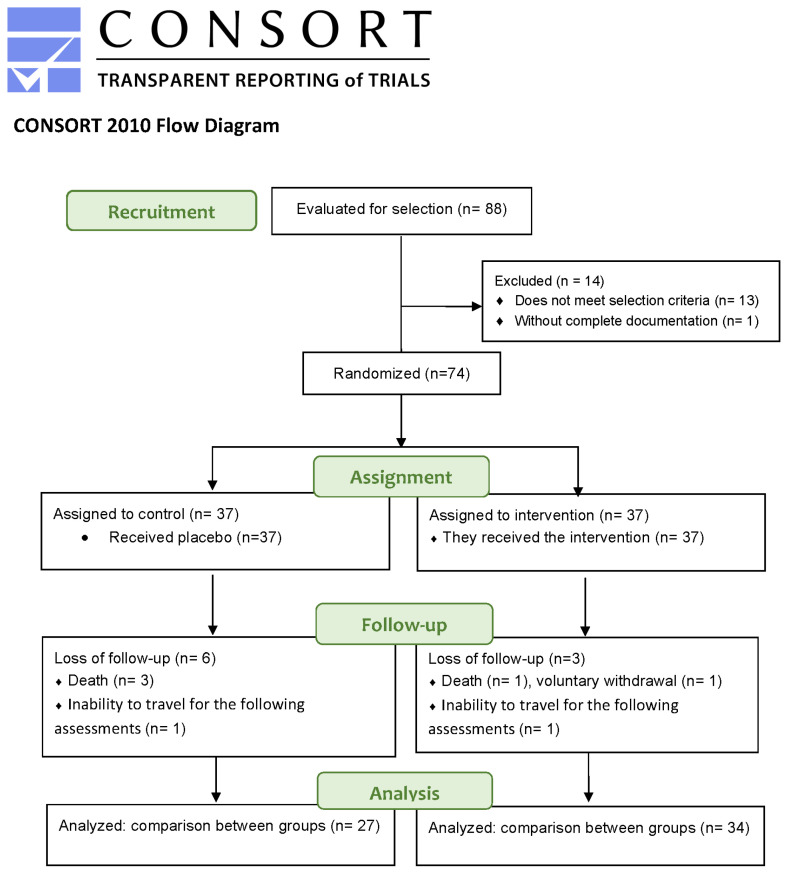
Flow diagram according to CONSORT.

The study was conducted at the IMED-UCV clinic in Valencia, Spain, between November 2021 and October 2022. All procedures followed the guidelines of the Declaration of Helsinki, and the study was approved by the Ethics Committee of the Hospital Universitario La Fe de Valencia, Spain (code 2021-001989 38). A CONSORT diagram is shown in Figure 6.

#### 4.3.1. Pharmacological Administration

The sample was randomly distributed into two groups: the intervention group (IG), which was administered curcumin in liposomal form/resveratrol^®^ aqueous solution (200 mg of curcuminoids (turmeric root) and 75 mg of resveratrol), and dutasteride (0.5 mg) in capsule form daily for 2 months. The control group (CG) was administered a placebo of water with sucrose, to which food coloring and thickener were added, thus achieving the same organoleptic characteristics as the antioxidant liposome, and a soft capsule of microcrystalline methylcellulose instead of dutasteride daily. The format of the container (the same bottles were used) and capsule (in terms of size, hardness, and color) was identical to that of the treatment administered to the IG.

All patients in the study were given administration guidelines for the daily intake of capsules and liquids. The baseline diet for all patients will be isocaloric and adapted to the nutritional needs of adults (approx. 2300 kcal/d in total). This diet will be personalized according to the metabolic and anthropometric characteristics of each patient and will not contain compounds such as those of the intervention (such as tea, coffee, or red fruits, which are rich in polyphenols). For the design of these diets, the software “Easydiet Programa de Gestión de la Consulta^®^” (Spanish Academy of Nutrition and Dietetics, Pamplona, Spain) was used (https://www.easydiet.es/, accessed 20 September 2021). They were instructed not to change the diet prescribed for each case, including how to cook the food, portion sizes, and products to buy in the supermarket.

#### 4.3.2. Determination of Functional Capacity

Functional capacity was measured in 61 patients using the ALS Functional Rating Scale-Revised (ALSFRS-R). This scale is sensitive, accurate, and reproducible and is used in patients with ALS to determine functional capacity, considering the domains of impairment: bulbar, upper limb, lower limb, and respiratory. The ALSFRS-R test consists of 12 items grouped into four domains that grade disabilities in activities of daily living. Each function is scored from 4 (normal) to 0 (no ability). Thus, the range of total functional disability varies from 0 (maximum disability) to 48 (normal) [72].

Electrical activity recording just before and after the 2 months of intervention—the recording of electrical activity was performed (performed by a single accredited specialist familiar with ALS patients, preserving intra- and inter-analytical reliability) by each of the study’s participants. For this purpose, with the subject in a seated position, the area where the electrode was to be placed was wiped dry using absorbent cotton to remove any possible sweat. With alcohol impregnated in another portion of this material, the area was cleaned and shaved with a disposable razor, leaving it free of possible interference with the electrode adhesive and distortion of the electrical signal owing to impedance. The area was wiped with alcohol and dried using absorbent cotton to clean and dry the skin.

Electrode placement: biceps brachii (BB): ventral medial and external part of the humerus, placed longitudinally; triceps brachii (TB): dorsal medial and internal part of the humerus, placed longitudinally; rectus femoris anterior (RF): central ventral portion of the femur, placed longitudinally; tibialis anterior (TA): 4 cm below the head of the fibula and longitudinally to it.

In all cases, two electrodes were placed with a maximum separation of 2 cm between them in the muscle belly, and a third (grounding) electrode was placed perpendicular to the previous electrodes.

The placement was bilateral, and the order by channel number was as follows: Channel 1, right BB; Channel 2, right TB; Channel 3, left BB; Channel 4, left TB; Channel 5, right RF; Channel 6, left RF; Channel 7, right AT; and Channel 8, left AT.

### 4.4. Instruments

**Figure 7:**
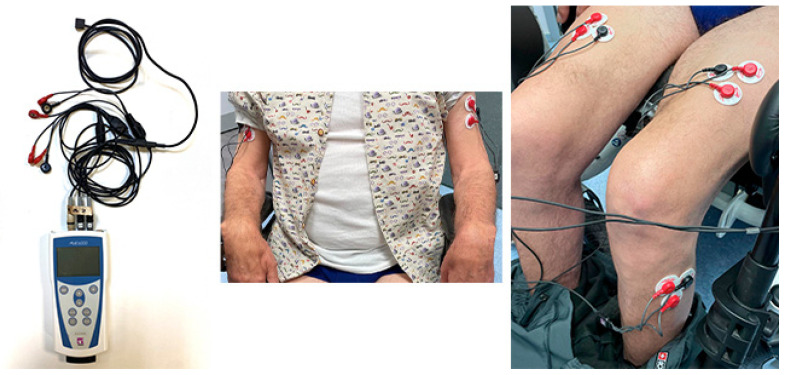
Placement of electrodes to obtain the sEMG signal. Note: (**left**) sEMG device; (**center**) electrode placement; global view; (**right**) lower limb electrode placement.

The electrodes used were of the Lessa Pediatric Electrode model, 30 mm in diameter, and positioned as indicated by SENIAM [73] and Criswell [74] (Figure 7).

To obtain the signal, an 8-channel Megawin ME6000-T8 model device (Bittium Corporation, Oulu, Finland) weighing 344 g was used. The sampling frequency was 1 kHz, and each of the stored files had a duration of 60 s with the subject seated and at rest.

After recording the data, they were converted from analog to digital (12-bit; DAQCard-700; National Instrument, Austin, TX, USA) using Biomonitor Megawin ME6000-T8 software and saved on a hard disk for protection and subsequent analysis.

MATLAB (R2024b) software (Mathworks Inc., Natick, MA, USA) was used for signal analysis. First, a fourth-order Butterworth bandpass filter between 20 and 400 Hz was applied to filter the signal and discard unspecific frequencies. Subsequently, the signal was rectified, or the Root Mean Square (RMS), by dividing the measurement section into 100 points. Finally, segmentation was performed by collecting 30 s of the signal in its most stable part.

The mean signal amplitude data as well as the smoothed mean with the smoothing function were collected to compare the recordings. In the case of fasciculations, the findpeaks function was used, modified to collect 10 peaks of activity in a central 30 s section with respect to the total time of the file, and ordered from greater to lesser activation for subsequent comparison in the different measurements.

### 4.5. Statistical Analysis

Statistical analyses were performed using SPSS version 23 software (IBM Corporation, Armonk, NY, USA). Descriptive data are shown as means and standard deviations for quantitative variables and as proportions for qualitative variables. The normal distribution of the variables was tested using the Kolmogorov–Smirnov test.

To compare the change between the initial measurements (t0) and at 2 months (t1) in each group, the Student’s *t*-test for paired samples or the Wilcoxon signed-rank test was used depending on whether the data met normality. The effect sizes were reported as Hedges’ g correction for Cohen’s d, as recommended for small samples. To compare the muscle activity in each group, Friedman’s test with Dunn’s test was used for multiple comparisons.

A significance level of 5% was used for all cases.

## 5. Conclusions

The main conclusion was that after the administration of curcumin in liposomal form, resveratrol, and dutasteride in patients with ALS, there was a change in muscle activation, mainly in the upper limb, which was accompanied by apparent stabilization of fasciculations. These findings seem to indicate that a combined treatment of curcumin and resveratrol liposomes together with the drug dutasteride clinically improves muscle function in ALS patients, which could in turn lead to an improvement in the quality of life in these patients.

The hypothesis of the study has been fulfilled, since this change has been produced not only by the increase in basal activation, but also by slight changes in fasciculations, more homogeneous in the IG with respect to that of the CG, opening an avenue of research that could continue to specify these aspects according to sex, type of ALS, and time of diagnosis.

These new data could broaden the experimental field with new therapies and lines of work for patients with ALS.

## Figures and Tables

**Table 1:** Descriptive data of the study sample.

		M ± SD
Age (years)		56.74 ± 10.47
	Women	38.2%
Diagnostic time (months)		29.63 ± 27.35
ALSFRS-R (points)	ALSFRS-R	29.16 ± 8.1
Type of ALS (by body region)	Spinal	80.9%
	Bulbar	19.1%

M ± SD: Mean and standard deviation for quantitative variables. Percentage (%) for categorical variables.

**Table 2:** Muscle activation in microvolts (μV) in the right and left hemifields.

	Control				Intervention			
	Pre	Post	*p* *	g	Pre	Post	*p* *	g
	M ± SD	M ± SD			M ± SD	M ± SD		
Total (μV)	34.32 ± 8.96	34.16 ± 12.51	0.041	0.01	21.31 ± 9.20	31.02 ± 14.59	0.035	−0.65
Total RH (μV)	15.45 ± 7.61	16.87 ± 7.36	0.489	−0.18	10.45 ± 5.51	13.90 ± 7.17	0.053	−0.45
Total LH (μV)	16.74 ± 7.9	16.60 ± 7.53	0.944	0.02	12.74 ± 6.32	21.54 ± 11	0.004	−0.74

Note: μV = Microvolts. RH, right hemibody; LH = Left hemibody; M = Mean; SD, standard deviation; *p* *, *p*-value from Student’s *t*-test in all cases; g = Hedges’ g correction for effect size.

**Table 3:** Muscle activation in microvolts (μV) of the highest peak value (P1) and lowest peak value (P10) in both groups at t0 and t1.

	Control						Intervention					
	Pre		Post		n	*p*-Value	Pre		Post		n	*p*-Value
	M	SD	M	SD			M	SD	M	SD		
BB-R P1	28.68	25.23	35.27	27.24	27	0.186	28.15	22.19	35.94	27.65	34	0.206
BB-R P10	14.41	12.31	12.65	9.90	27	0.548	12.23	12.67	14.20	13.54	32	0.011
BB-L P1	19.27	14.81	22.80	17.16	27	0.968	17.13	11.06	30.81	23.33	33	0.056
BB-L P10	8.82	6.83	9.21	5.00	26	0.710	8.72	6.16	11.49	8.67	29	0.220
TB-R P1	23.79	16.57	19.67	12.31	27	0.209	18.78	14.89	19.71	17.08	30	0.246
TB-R P10	10.32	8.55	9.29	6.43	26	0.511	8.70	7.55	9.11	8.65	32	0.452
TB-L P1	23.77	17.71	32.65	27.99	26	0.424	25.47	21.16	35.40	23.09	29	0.012
TB-L P10	12.87	9.77	14.48	11.21	26	0.797	12.04	9.77	18.15	14.66	32	0.007
Total upper	483.79	244.07	585.23	295.34	22	0.477	446.07	243.69	611.73	374.29	22	0.017
RF-R P1	9.00	5.69	17.56	14.04	26	0.001	11.25	9.03	20.81	18.54	32	0.003
RF-R P10	4.90	3.76	7.38	5.94	27	0.055	5.34	4.47	7.66	7.33	31	0.076
RF-L P1	12.25	8.89	13.65	8.87	26	0.501	13.98	10.43	16.98	14.01	32	0.313
RF-L P10	3.1	2.40	6.38	5.19	26	0.004	5.33	4.41	8.12	6.25	31	0.118
TA-R P1	17.56	14.81	19.69	18.04	25	0.710	16.53	12.11	16.82	16.30	32	0.381
TA-R P10	4.37	3.96	5.28	4.38	25	0.689	5.87	4.43	4.42	3.93	30	0.265
TA-L P1	12.62	9.45	20.90	18.27	27	0.036	20.45	18.33	17.02	14.26	34	0.281
TA-L P10	7.03	6.31	4.90	3.79	23	0.372	7.36	7.22	5.02	4.36	33	0.240
Total lower	233.10	96.71	327.11	172.03	17	0.037	275.26	165.74	315.57	177.53	24	0.535

Note: M, mean value; SD = Standard deviation; BB = Biceps brachii; TB = Triceps brachii; RF = Rectus femoris; TA = Tibialis anterior; P1 = Peak 1; P10 = Peak 10; t0, time/measurement 0; t1, time/measurement 1.

## Data Availability

The datasets used and analyzed during the current study are available from the corresponding author upon reasonable request, owing to privacy and ethical restrictions.

## Abbreviations

The following abbreviations are used in this manuscript:

DHP	Dihydroprogesterone
DHT	Dihydrotestosterone
ALS	Amyotrophic Lateral Sclerosis
ND	Neurodegenerative diseases
CG	Control group
IG	Intervention group
PROG	Progesterone
T	Testosterone
BB	Biceps brachii
TB	Triceps brachii
RF	Rectus femoris
TA	Tibialis anterior
sEMG	surface electromyography

## Appendix A

**Table A1:** Comparison of muscle groups at time 0 and 1 in both experimental groups.

	CONTROL						INTERVENTION					
	M ± SD	Me [RIC]	N	Min	Max	*p*-Value	M ± SD	Me [RIC]	N	Min	Max	*p*-Value
Mean BB R T0	6.71 ± 5.6	5.65 [4.92]	18	1.33	20.87	<0.001	4.1 ± 3.64	2.84 [3.85]	18	1.04	15.46	0.003
Mean TB R T0	3.49 ± 2.57	3.06 [3.06]	18	0.94	12		2.79 ± 1.63	2.28 [1.69]	18	0.8	6.07	
Mean RF R T0	2.16 ± 1.24	1.72 [1.72]	18	0.83	4.9		1.7 ± 1.06	1.43 [0.7]	18	0.68	5.18	
Mean TA R T0	2.14 ± 1.5	1.54 [1.22]	18	0.96	5.96		2.65 ± 2.17	1.89 [2.2]	18	0.74	9.96	
Mean BB L T0	3.96 ± 2.71	2.9 [2.89]	18	1.11	11.15		3.79 ± 3.33	2.81 [2.93]	18	1.03	13.69	
Mean TB L T0	6.09 ± 4.01	5.1 [6.38]	18	1.23	13.89		4.97 ± 4.43	3.5 [5.36]	18	1.26	16.34	
Mean RF L T0	1.96 ± 1.15	1.44 [1.85]	18	0.8	4.85		2.07 ± 1.44	1.38 [1.82]	18	0.79	5.84	
Mean TA L T0	2.81 ± 1.85	1.99 [1.95]	18	0.87	7.01		2.17 ± 1.21	1.83 [1.73]	18	0.83	4.97	
Mean BB R T1	8.04 ± 6.58	5.36 [12.88]	16	1.27	20.71	<0.001	5.65 ± 5.48	3.44 [6.76]	18	0.83	17.87	0.017
Mean TB R T1	4.87 ± 3.93	4.62 [3.94]	16	1.18	17.69		3.31 ± 2.15	2.67 [4.02]	18	0.9	6.77	
Mean RF R T1	2.27 ± 1.61	1.8 [2.03]	16	0.8	6.69		2.23 ± 1.28	2.18 [1.56]	18	0.64	5.81	
Mean TA R T1	3.3 ± 2.36	2.25 [3.54]	16	0.74	9.28		2.03 ± 1.24	1.83 [1.51]	18	0.79	5.58	
Mean BB L T1	4.75 ± 2.92	4 [4.97]	16	1.39	10.65		4.72 ± 3.7	3.89 [5.09]	18	1.09	13.52	
Mean TB L T1	7.33 ± 6.22	3.7 [10.95]	16	0.82	19.15		6.24 ± 6.84	3.72 [7.12]	18	1.05	28.02	
Mean RF L T1	2.72 ± 1.13	2.81 [1.05]	16	0.89	5.57		2.95 ± 2.26	2.46 [2.8]	18	0.71	8.72	

Note: M: mean value; SD = Standard deviation; BB = Biceps brachii; TB = Triceps brachii; RF = Rectus femoris; TA = Tibialis anterior; T0: time/measurement 0; T1: time/measurement 1; L = Left; R = Right.

**Table A2:** Effect sizes for paired comparisons of basal and fasciculation measurements in the control and intervention groups.

	Control		Intervention	
	Hedges’ g	IC 95%	Hedges’ g	IC 95%
Basal activation				
Total basal	0.012	(−0.58; 0.6)	−0.647	(−1.23; −0.05)
Total RH	−0.179	(−0.67; 0.32)	−0.448	(−0.95; 0.07)
Total LH	0.016	(−0.43; 0.46)	−0.743	(−1.25; −0.21)
BB R	−0.152	(−0.52; 0.22)	−0.375	(−0.77; 0.02)
TB R	−0.141	(−0.54; 0.26)	0.086	(−0.28; 0.45)
RF R	−0.072	(−0.47; 0.33)	−0.262	(−0.64; 0.12)
TA R	−0.135	(−0.54; 0.27)	0.171	(−0.17; 0.51)
BB L	−0.172	(−0.57; 0.23)	−0.385	(−0.76; 0)
TB L	−0.092	(−0.48; 0.3)	−0.39	(−0.76; −0.02)
RF L	−0.521	(−0.92; −0.12)	−0.389	(−0.76; −0.01)
TA L	0.443	(0.03; 0.85)	0.339	(−0.04; 0.71)
Fasciculations				
BB R P1	−0.205	(−0.48; 0.07)	−0.238	(−0.66; 0.19)
BB R P10	0.205	(−0.25; 0.66)	−0.362	(−0.74; 0.01)
BB L P1	−0.005	(−0.47; 0.46)	−0.584	(−1.1; −0.07)
BB L P10	0.058	(−0.46; 0.57)	−0.46	(−1.01; 0.09)
TB R P1	0.377	(−0.12; 0.87)	−0.201	(−0.7; 0.3)
TB R P10	0.204	(−0.19; 0.6)	−0.087	(−0.49; 0.32)
TB L P1	−0.172	(−0.6; 0.25)	−0.519	(−1.02; −0.02)
TB L P10	−0.045	(−0.61; 0.52)	−0.526	(−0.91; −0.14)
RF R P1	−0.638	(−1.1; −0.17)	−0.499	(−0.81; −0.19)
RF R P10	−0.458	(−0.87; −0.05)	−0.299	(−0.67; 0.07)
RF L P1	−0.105	(−0.57; 0.36)	−0.203	(−0.51; 0.11)
RF L P10	−0.586	(−1.1; −0.08)	−0.383	(−0.92; 0.15)
TA R P1	−0.143	(−0.61; 0.33)	0.049	(−0.3; 0.4)
TA R P10	−0.181	(−0.87; 0.51)	0.272	(−0.12; 0.67)
TA L P1	−0.446	(−0.83; −0.07)	0.202	(−0.23; 0.63)
TA L P10	0.314	(−0.26; 0.89)	0.312	(−0.13; 0.76)
Total Upper	−0.04	(−0.96; 0.88)	−0.858	(−1.56; −0.15)
Total Lower	−0.68	(−1.44; 0.08)	−0.141	(−0.69; 0.4)

Note:; BB = Biceps brachii; TB = Triceps brachii; RF = Rectus femoris; TA = Tibialis anterior; T0: time/measurement 0; T1: time/measurement 1; L = Left; R = Right.
